# High-intensity interval training reduced oxidative stress and apoptosis in the hippocampus of male rats with type 2 diabetes: The role of the PGC1α-Keap1-Nrf2 signaling pathway

**DOI:** 10.22038/IJBMS.2023.70833.15387

**Published:** 2023

**Authors:** Narjes Ebrahimnezhad, Shila Nayebifar, Zahra Soltani, Kayvan Khoramipour

**Affiliations:** 1 Department of Sports Science, Faculty of Educational Sciences and Psychology, Sistan and Baluchestan University, Zahedan, Iran; 2 Endocrinology and Metabolism Research Center, Institute of Basic and Clinical Physiology Sciences, Afzalipour Faculty of Medicine, Kerman University of Medical Sciences, Kerman, Iran; 3 Student Research Committee, Afzalipour Faculty of Medicine, Kerman University of Medical Sciences, Kerman, Iran

**Keywords:** Anti-oxidant enzymes, Apoptosis, Hippocampus, PGC1α, Type 2 diabetes

## Abstract

**Objective(s):**

This study aimed to determine the effect of 8-week high-intensity interval training (HIIT) on oxidative stress and apoptosis in the hippocampus of male rats with type 2 diabetes (T2D). The study focused on examining the role of proliferator-activated receptor gamma co-activator 1α (PGC1α)/Kelch-like ECH-associated protein Keap1/nuclear factor erythroid 2-related factor 2 (Nrf2) signaling pathway.

**Materials and Methods:**

Twenty-eight 8-week-old Wistar rats were randomly assigned to one of four groups (n=7): control (Con), type 2 diabetes (T2D), exercise (Ex), and exercise + type 2 diabetes (Ex+T2D). The Ex and Ex+T2D groups completed an 8-week exercise program consisting of 80-100% Vmax and 4–10 intervals. The homeostasis model assessment of insulin resistance (HOMA-IR) index was used to assess insulin resistance. The levels of Bcl2, BAX, musculoaponeurotic fibrosarcoma (Maf), Nrf2, Keap1, and PGC1α in the hippocampus were assessed using the western blot method. Additionally, the levels of antioxidant enzymes in the hippocampus were measured using ELISA.

**Results:**

The findings indicated that the T2D group had lower levels of antioxidant enzymes, Maf, Bcl2, PGC1α, and Nrf2, and higher levels of BAX and Keap1 in the hippocampus. Conversely, the HIIT group exhibited increased levels of antioxidant enzymes, Maf, Bcl2, Nrf2, and PGC1α, along with decreased levels of BAX and Keap1 in the hippocampus.

**Conclusion:**

The study demonstrated that 8-week HIIT was effective in reducing hippocampal apoptosis and oxidative stress induced by T2D by activating the PGC1α-Keap1-Nrf2 signaling pathway. The metabolic changes induced by exercise may lead to an increase in PGC1 expression, which is the primary stimulator of the Keap1-Nrf2 signaling pathway.

## Introduction

Type 2 diabetes (T2D) is a chronic metabolic disorder characterized primarily by hyperglycemia and insulin resistance (IR) (1, 2). The prevalence of T2D has increased due to the modern socio-economic lifestyle, which has reduced activity and increased fast food consumption, reaching epidemic proportions worldwide (3). The global prevalence of T2D is expected to reach 592 million by 2035 (4, 5). The associated health costs of diabetes were estimated to be $966 billion in 2021 and are projected to reach $1,054 billion by 2045 (6).

Oxidative stress is a major causative factor and consequence of diabetes (7). Oxidative stress has been found in the hippocampus and peripheral organs of T2D patients (3, 8). A study (9) demonstrated that oxidative stress in the hippocampus can impair its normal function and lead to cognitive impairments. Reactive oxygen species (ROS)-induced oxidative stress can damage the cell membrane and adversely affect biological molecules such as proteins, lipids, and nucleic acids in the brain, leading to apoptosis (4). Apoptosis is a programmed cell death that is modulated by the Bcl-2 family proteins, which are classified into those that enhance cell survival, such as Bcl-2, and those that promote cell death, such as Bax (10). The Bax/Bcl2 ratio is used as an apoptosis indicator (11). Researchers (12) reported that streptozotocin (STZ) injection increases oxidative stress and apoptosis in the hippocampus, resulting in a reduction in spatial memory. Moreover, another study reported a clear association between IR, oxidative stress, and memory impairments in T2D (13).

Peroxisome proliferator-activated receptor-gamma coactivator 1-alpha (PGC-1α) is a transcription factor that plays crucial roles in mitochondrial biogenesis, regulation of mitochondrial function, and detoxification of ROS (14, 15). In a study (15) on obese rats with T2D, a decrease in PGC-1α protein expression and impaired mitochondrial function were observed. This suggests that a reduction in PGC-1α levels could lead to an increase in ROS and ultimately result in memory impairments (16, 17). PGC-1α is responsible for controlling the expression of anti-oxidant genes by activating the Keap1-Nrf2-ARE signaling pathway (18). Keap1 is an adaptor protein dimer that consists of 624 amino acids and is responsible for cullin3-based ubiquitin E3 ligase. Keap1 suppresses the activity of nuclear factor erythroid 2-related factor 2 (Nrf2). Under normal conditions, Keap1 facilitates the degradation and ubiquitination of Nrf2 using the ubiquitin-proteasome pathway, thereby inhibiting Nrf2 signaling by preventing its transportation to the nucleus (19). However, when cells experience oxidative stress, Keap1 cysteine residues are chemically modified. This modification prevents Nrf2 protein degradation and ubiquitination, leading to Nrf2 stabilization and its accumulation in the nucleus. Once in the nucleus, Nrf2 forms a heterodimer with small Maf proteins and binds to the anti-oxidant response element (ARE) sequence located in the promoter of anti-oxidant genes, thereby facilitating transcription (20). The Keap1-Nrf2 mechanism regulates gene response to stress and is an essential element in the regulation of protective responses to ROS (20). Under normal conditions, there is a balance between free radicals and anti-oxidant enzyme levels, which is necessary for biological homeostasis (21, 22). However, when free radical species are excessively generated in the body, a distortion in the balance with the body’s anti-oxidant capacity leads to oxidative stress. This can occur when the production of free radicals exceeds physiological levels, which overloads the cell anti-oxidant systems, causing oxidative stress (23). Free radicals have a toxic effect when the body’s anti-oxidant protective mechanisms are overwhelmed by excessive free radical generation, which leads to oxidative stress (24).

Oxidative stress is one of the important upstreams in the development of IR and complications of diabetes (25). Exercise has been proven to be one of the most effective non-pharmacological methods of reducing oxidative stress and apoptosis. Endurance training has been shown to reduce oxidative stress by increasing mitochondrial biogenesis (26) and activating PGC-1α (27). Ran *et al*. (28) showed that hippocampal neuron PGC-1α expression increased after 2 weeks of endurance training. The effect of exercise on oxidative stress shows a dose-response pattern, and intensity is considered the most important factor affecting oxidative stress adaptation following exercise training. Consequently, high-intensity interval training (HIIT) is considered an excellent option for improving oxidative status (29). Therefore, it was hypothesized that HIIT could improve oxidative stress and apoptosis induced by T2D in the hippocampus. This study aimed to investigate the effect of 8 weeks of HIIT on the PGC-1α-Keap1-Nrf2 signaling pathway, oxidative stress, and apoptosis in the male rats hippocampus with T2D.

## Materials and Methods

***Animal care***

A total of twenty-eight male Wistar rats, aged 8 weeks and weighing 200 g, were obtained from Kerman University of Medical Sciences (KUM) animal farm to be used in the study. The rats were housed in specialized polycarbonate cages and maintained in a controlled environment with a 12-hour light-dark cycle, *ad libitum* access to food and water, and a temperature of 23 ± 2 °C. The study protocol was approved by the KUM Ethics Committee (ethics code ir.kmu.aec.1401.003). Following habituation to the environment, the rats were randomly divided into four groups with ten rats in each group: control (Con), diabetes (T2D), exercise (Ex), and T2D+exercise (T2D+Ex). The Ex and T2D+Ex groups underwent an eight-week high-intensity interval training (HIIT) program.

***Diabetes induction***

The T2D and T2D+Ex groups were subjected to a two-month high-fat diet (HFD) as shown in Table 1. After the end of the two-month diet, the animals were fasted for 12 hr and then given a single intraperitoneal dose of streptozotocin at a concentration of 35 mg/kg. Three days after the administration of STZ, blood glucose levels were checked using a glucometer (Accu-Chek, USA). Diabetic rats (fasting blood glucose (FBG) > 300 mg/dl) were included in the study (3).

***Exercise protocol***

Prior to the start of the experiments, all animals were given a five-day familiarization period with the motorized treadmill, during which they ran on it for 10 min per day at a speed of 8 meters per minute and an incline of 0%. After this period, their Vmax was determined, and they participated in an 8-week high-intensity interval training (HIIT) program developed in our laboratory, which we referred to as the “k1 protocol.” The details of this protocol have been explained in our previous publication, and it involved a 5 min warm-up and cool-down period at 40–50% of Vmax (3, 17).

***Tissue sampling***

After the completion of the final training session, the animals were anesthetized 48 hr later using a combination of ketamine (80 mg/kg) and xylazine (10 mg/kg). The study variables were assessed using the hippocampal tissue.

***Western blot***

Western blotting was used to measure the amount of PGC1α (Anti-PGC1 alpha antibody ab54481), KEAP1 (SANTA CRUZ BIOTECHNOLOGY, INC, sc-365626), Nrf2 (SANTA CRUZ BIOTECHNOLOGY, INC, sc-365949), Maf (SANTA CRUZ BIOTECHNOLOGY, INC, sc-166806), BAX (SANTA CRUZ BIOTECHNOLOGY, INC, sc-7480), and BCL2 (SANTA CRUZ BIOTECHNOLOGY, INC, sc-492). For this purpose, the hippocampal total protein concentration was measured using the Lowry method, with bovine serum albumin as the standard. Forty micrograms of protein from each sample was added to a buffer sample, after matching the concentrations. The mixture was then subjected to 75 min electrophoresis using 11% SDS-PAGE gel. The resulting gel, containing the separated proteins, was transferred to PVDF paper. This was followed by overnight incubation of the membrane at 4 °C in a 2% block solution. The membrane was then washed four times with TBST solution for 5 min each, and subsequently incubated for 3 hr with the primary antibody (1:200 concentration) of each protein, and then for 1 hr with the secondary antibody (1:1000 concentration). Finally, immune detection was recorded using the Chemi Doc XRS+ imaging system (Bio-Rad Company, USA), and the results were analyzed using the ImageJ software package (30), with β-actin serving as the control.

***ELISA***

ELISA was employed to measure the concentrations of insulin and anti-oxidant enzymes (i.e. GPX, SOD, and CAT) in the hippocampus supernatant. The anti-oxidant enzymes (ZellBio GmbH, ZB-SOD-96A, ZB-CAT-96A, ZB-GPX-96A) and insulin (Rat ELISA Kit, Eastbiopharm) were assayed using appropriate kits.

***Calculation of insulin resistance*** ***index***

IR was evaluated using the homeostasis model assessment (HOMA), and specifically, the HOMA-IR score was calculated using the following formula: HOMA-IR = [(fasting glucose (mmol/l) × fasting insulin (μU/ml)) / 22.5] (31).

***Statistical analysis***

The data were expressed as mean±standard deviation (SD). Statistical analysis was conducted using SPSS version 21. The homogeneity and normality of variances were evaluated using the Leven and Shapiro-Wilk tests, respectively. Two-way ANOVA was performed followed by Tukey *post hoc* test. A *P*-value<0.05 was considered statistically significant.

## Results

***Animal blood glucose and weight***

**Figure 1:**
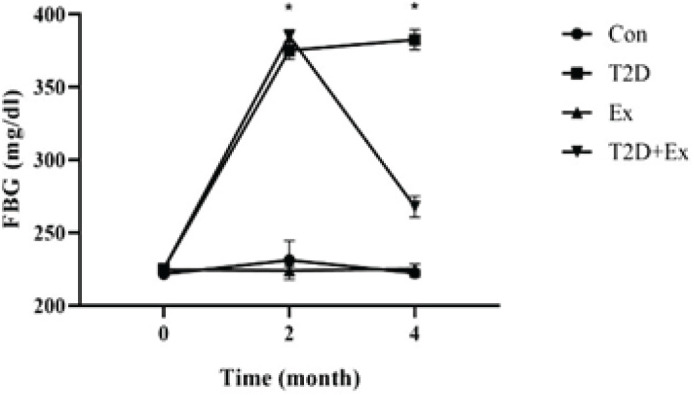
Fasting blood glucose levels (mean±SD) at three time points (i.e., before the start of the intervention (month 0), after diabetes induction (2 months of high-fat diet and STZ injection) (month 2), and 48 hr after the final training session (month 4)) in all groups (n= 7 in each group)

To confirm the effectiveness of our diabetes induction method, we evaluated FBG. The results revealed a significant increase in FBG levels after diabetes induction (2 months of high-fat diet and STZ injection) as compared to the baseline (month 0), in both the T2D and T2D+Ex groups (*P*=0.000). There was no significant difference in FBG levels between these two groups. Furthermore, the HIIT intervention resulted in a significant reduction in FBG (*P*=0.000), (Figure 1).

**Figure 2:**
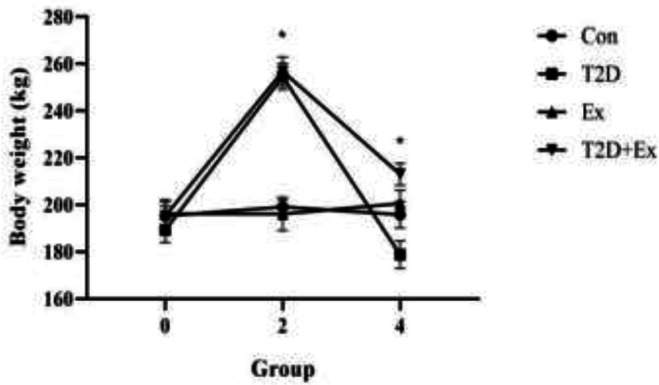
Weight (mean±SD) at four different time points (i.e., before the start of the intervention (month 0), after diabetes induction (2 months of high-fat diet and STZ injection) (month 2), and 48 hr after the final training session (month 4)) in all groups (n=7 in each group)

Following diabetes induction (2 months of high-fat diet and STZ injection), there was a significant increase in animal weight in the T2D and T2D+Ex groups (*P*=0.000). Subsequently, there was a decrease in weight observed in both these groups (*P*=0.000), with a greater decrease seen in the T2D group (*P*=0.02) (Figure 2).

***Insulin resistance index***

**Figure 3:**
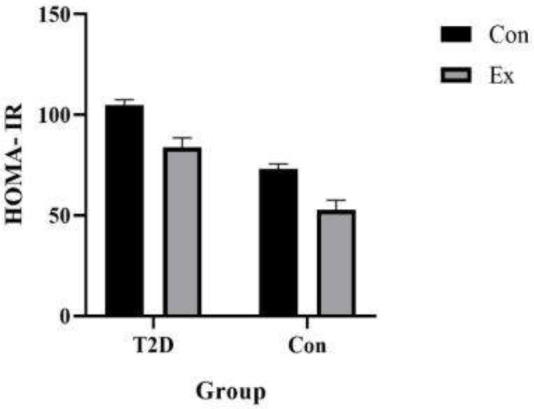
Homeostatic Model Assessment for Insulin Resistance (HOMA-IR) (n=7 in each group)

To determine whether exercise could improve insulin sensitivity, we assessed the insulin sensitivity index (i.e., HOMA-IR). Our findings indicate that HOMA-IR increased in the T2D group and decreased in the Ex group (*P*<0.05). Furthermore, a significant interaction was observed between T2D and Ex (*P*<0.05) (Figure 3).

**Figure 4:**
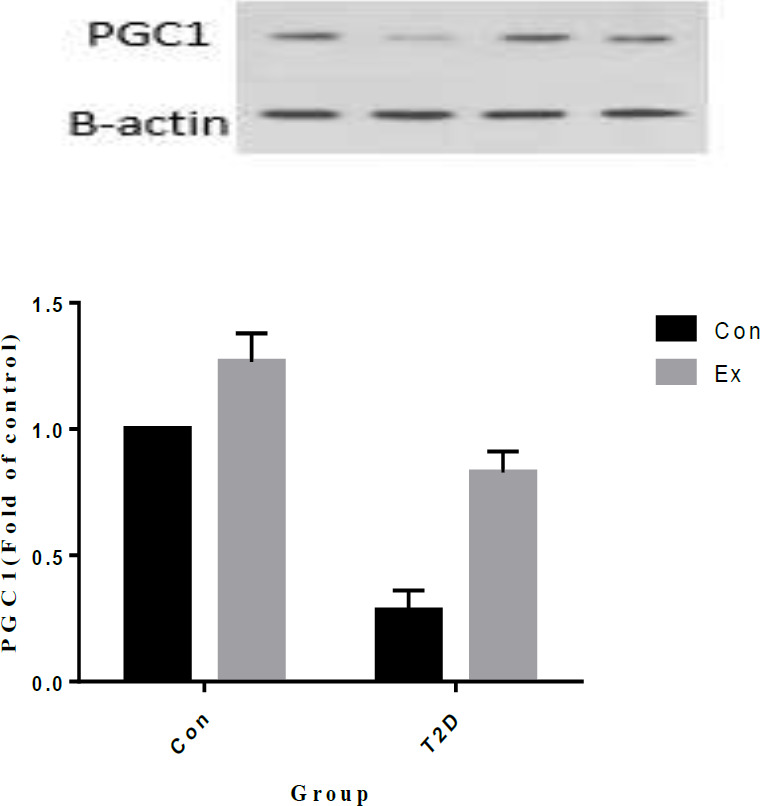
Peroxisome proliferator-activated receptor-gamma coactivator-1alpha (PGC1α) levels (mean±SD) in the hippocampus (n=7 in each group)

Our study results revealed that T2D led to a significant decrease in PGC1α levels, while Ex resulted in a significant increase in PGC1α levels (*P*<0.05). Additionally, there was a significant interaction observed between T2D and Ex (*P*<0.05) (Figure 4).

**Figure 5:**
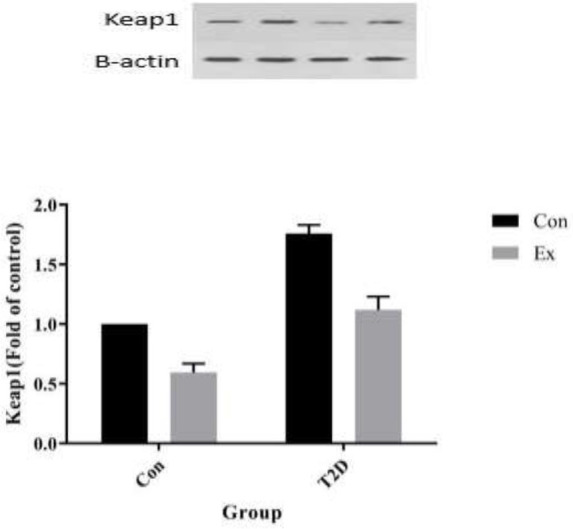
Kelch-like ECH-associated protein 1 ( Keap1) levels (mean±SD) in the hippocampus (n=7 in each group)

The analysis of our findings revealed that T2D led to a significant increase in Keap1 levels in the hippocampus, while Ex resulted in a significant decrease in Keap1 levels (*P*<0.05). Furthermore, there was a significant interaction observed between T2D and Ex (*P*<0.05) (Figure 5).

**Figure 6:**
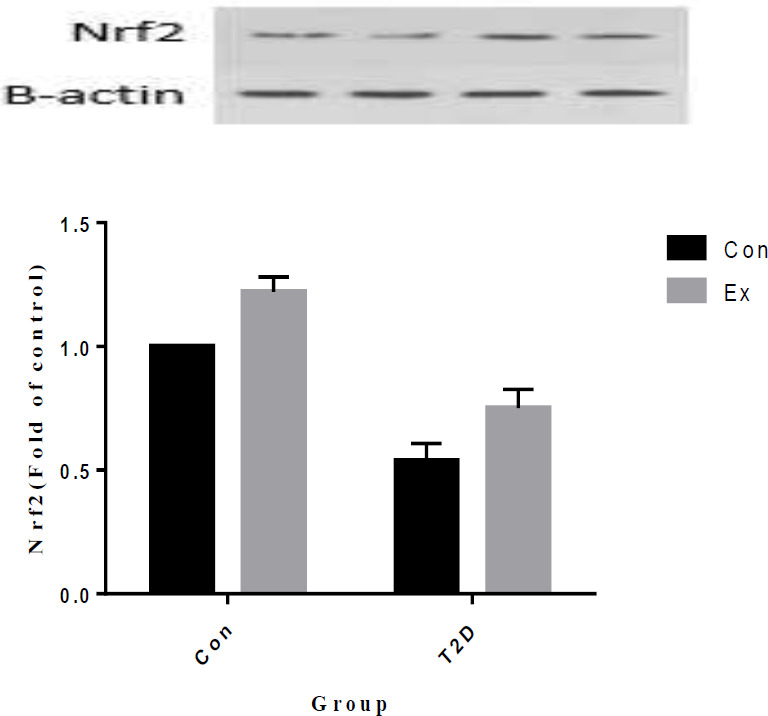
Nuclear factor erythroid 2–related factor 2 (Nrf2) levels (mean±SD) in the hippocampus (n=7 in each group)

Our study results indicate that T2D led to a significant decrease in Nrf2 levels in the hippocampus, while Ex resulted in a significant increase in Nrf2 levels (*P*<0.05). Additionally, there was a significant interaction observed between T2D and Ex (*P*<0.05) (Figure 6).

**Figure 7:**
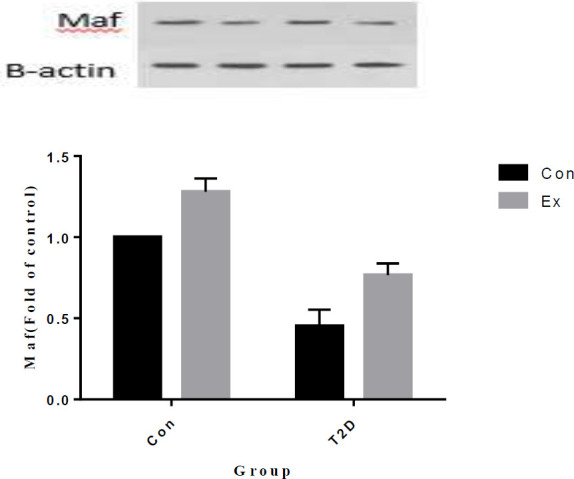
Maf levels (mean±SD) in the hippocampus (n=7 in each group)

The analysis of our findings revealed that T2D led to a significant decrease in Maf levels in the hippocampus, while Ex resulted in a significant increase in Maf levels (*P*<0.05). Furthermore, there was a significant interaction observed between T2D and Ex (*P*<0.05) (Figure 7).

**Figure 8:**
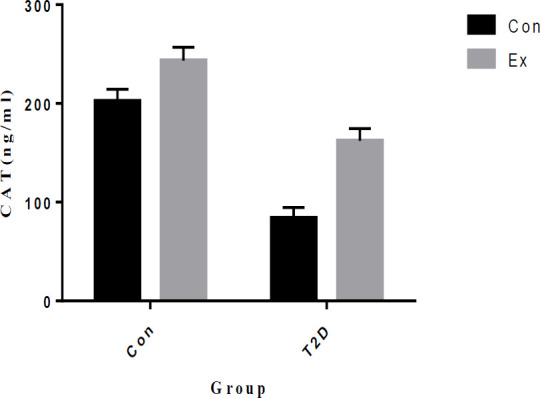
Catalase (CAT) levels (mean±SD) in the hippocampus (n=7 in each group)

Our study findings indicate that T2D led to a significant decrease in CAT levels in the hippocampus, while Ex resulted in a significant increase in CAT levels (*P*<0.05). Additionally, there was a significant interaction observed between T2D and Ex (*P*<0.05) (Figure 8).

**Figure 9:**
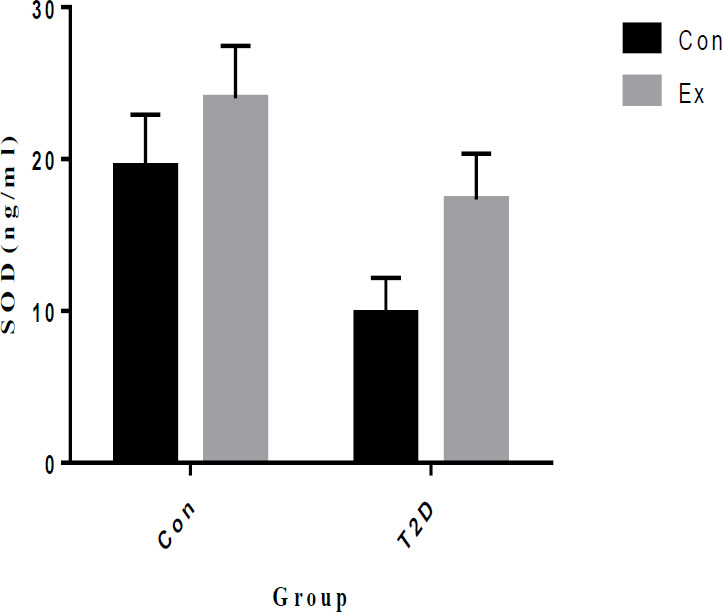
Superoxide dismutase (SOD) levels (mean±SD) in the hippocampus (n=7 in each group)

Our study results indicate that T2D led to a significant decrease in SOD levels in the hippocampus, while Ex resulted in a significant increase in SOD levels (*P*<0.05). Additionally, there was a significant interaction observed between T2D and Ex (*P*<0.05) (Figure 9).

**Figure 10:**
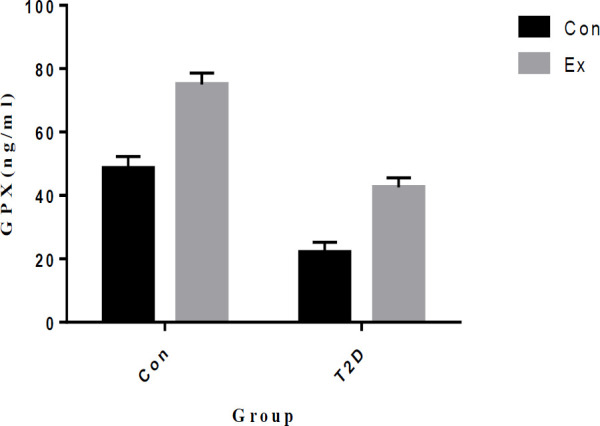
Glutathione peroxidase (GPX) levels (mean±SD) in the hippocampus (n=7 in each group)

Our study findings reveal that T2D led to a significant decrease in GPX levels in the hippocampus, while Ex resulted in a significant increase in GPX levels (*P*<0.05). Furthermore, there was a significant interaction observed between T2D and Ex (*P*<0.05) (Figure 10).

**Figure 11:**
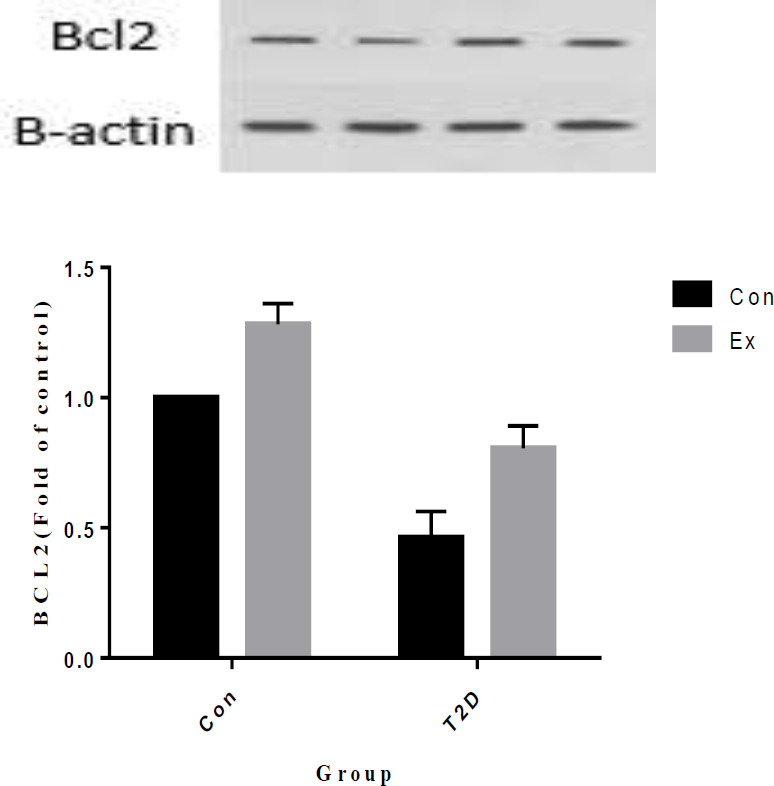
Bcl2 levels (mean±SD) in the hippocampus (n=7 in each group)

The analysis of our findings indicates that T2D led to a significant decrease in BCL2 levels in the hippocampus, while Ex resulted in a significant increase in BCL2 levels (*P*<0.05). Additionally, there was a significant interaction observed between T2D and Ex (*P*<0.05) (Figure 11).

**Figure 12:**
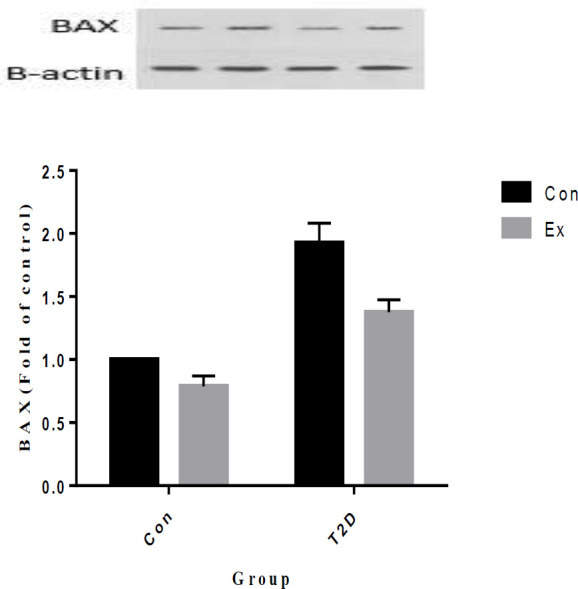
BAX levels (mean±SD) in the hippocampus (n=7 in each group)

Our study results indicate that T2D led to a significant increase in BAX levels in the hippocampus, while Ex resulted in a significant decrease in BAX levels (*P*<0.05). Moreover, there was a significant interaction observed between T2D and Ex (*P*<0.05) (Figure 12).

**Figure 13:**
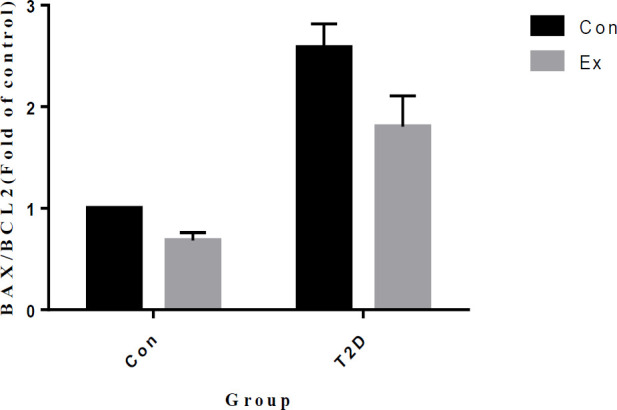
BAX/Bcl2 levels (mean±SD) in the hippocampus (n=7 in each group)

The analysis of our findings indicates that T2D led to a significant increase in BAX/BCL2 levels in the hippocampus, while Ex resulted in a significant decrease in BAX/BCL2 levels (*P*<0.05). Additionally, there was a significant interaction observed between T2D and Ex (*P*<0.05)(Figure 13).

FBG: Fasting blood glucose, Con: control, T2D: Type 2 diabetic (STZ injected), Ex: exercise only, and T2D+Ex: Type 2 diabetic + exercise. * Shows a significant difference between T2D and T2D+Ex with other groups

Con: control, T2D: Type 2 diabetic, Ex: exercise, and T2D+Ex: Type 2 diabetic+ exercise. * Shows a significant difference between T2D with other groups

Con: Control; T2D: Type 2 diabetic, Ex: Exercise

T2D effect: *P*<0.05, Ex effect: *P*<0.05, Interaction: *P*<0.05

Con: Control, T2D: Type 2 diabetic, Ex: Exercise

T2D effect: *P*<0.05, Ex effect: *P*<0.05, Interaction: *P*<0.05

Con: Control; T2D: Type 2 diabetic; Ex: Exercise

T2D effect: *P*<0.05, Ex effect: *P*<0.05, Interaction: *P*<0.05

Con: Control, T2D: Type 2 diabetic, Ex: Exercise

T2D effect:* P*<0.05, Ex effect: *P*<0.05, Interaction: *P*<0.05

Con: Control, T2D: Type 2 diabetic, Ex: Exercise

T2D effect: *P*<0.05, Ex effect: *P*<0.05, Interaction: *P*<0.05

Con: Control, T2D: Type 2 diabetic, Ex: Exercise

T2D effect: *P*<0.05, Ex effect: *P*<0.05, Interaction: *P*<0.05

Con: Control, T2D: Type 2 diabetic, Ex: Exercise

T2D effect: *P*<0.05, Ex effect: *P*<0.05, Interaction: *P*<0.05

Con: Control, T2D: Type 2 diabetic, Ex: Exercise

T2D effect: *P*<0.05, Ex effect: *P*<0.05, Interaction: *P*<0.05

Con: Control, T2D: Type 2 diabetic, Ex: Exercise

T2D effect: *P*<0.05, Ex effect: *P*<0.05, Interaction: *P*<0.05

Con: Control, T2D: Type 2 diabetic, Ex: Exercise

T2D effect: *P*<0.05, Ex effect: *P*<0.05, Interaction: *P*<0.05

Con: Control, T2D: Type 2 diabetic, Ex: Exercise

T2D effect: *P*<0.05, Ex effect: *P*<0.05, Interaction: *P*<0.05

## Discussion

The present study aimed to investigate the effects of 8 weeks of HIIT on the PGC1α-Keap1-Nrf2 signaling pathway, the Bax/Bcl2 ratio, and the levels of anti-oxidant enzymes (i.e., SOD, CAT, GPX) in the hippocampus of male rats with T2D. However, HIIT increased the levels of PGC1α, Nrf2, Maf, Bcl2, and anti-oxidant enzymes in the hippocampal region of T2D rats, but the levels of Keap1 and Bax decreased.

A previous study has shown that increased oxidative stress and mitochondrial dysfunction in the hippocampus are among the most significant causes of memory disorders in diabetic patients (32). PGC1α has been identified as a potential therapeutic target due to its crucial role in regulating mitochondrial biogenesis and resistance to oxidative stress. PGC1α also regulates the expression of anti-oxidants via the Keap1-Nrf2 pathway (33). The present study findings are consistent with previous research (34) that has reported a decrease in PGC1α levels in diabetic rats. IR, which is common in T2D, can increase ROS production and oxidative stress (35), resulting in decreased PGC1α expression (36). However, the present study also found that 8 weeks of HIIT increased PGC1α levels in the hippocampal region, which is consistent with previous studies by Zoladz *et al*. (37) and Steiner *et al*. (38). Zoladz *et al*. reported that eight weeks of endurance training, five times per weeks, increased the oxidative capacity in rats. Furthermore, a study (39) demonstrated that 6 weeks of HIIT and endurance training resulted in metabolic adaptations and an increase in PGC-1α levels, which is consistent with the results of the present study. However, Ikeda *et al*. (40) and Little *et al*. (41) did not observe significant changes in muscular levels of PGC-1α after exercise training. This may be due to the insufficient training intensity (voluntary wheel running) in Ikeda *et al*.’s study and the short duration of the training protocol (two weeks) in Little’s study. Additionally, while we measured PGC-1α levels in the hippocampus, Ikeda *et al*. and Little *et al*. measured it in muscles. 

The exact mechanism by which exercise increases PGC-1α expression and promotes adaptation is not yet clear. However, several mechanisms have been suggested, including the calcineurin A, Ca²⁺/calmodulin-dependent protein kinase (CaMK), p38 mitogen-activated protein kinases (p38MAPK), and AMP-activated protein kinase (AMPK) pathways (42). Among these pathways, the calcineurin A and CaMK pathways appear to be more important because exercise-induced neuromuscular input and muscle contraction stimulate the expression of various transcription factors, such as the myocyte enhancer factor 2 (MEF2) and cAMP response element-binding protein (CREB), which are induced by calcineurin and CaMK (43). This leads to an increase in PGC-1α expression (44). The increase in PGC-1α levels stimulates the Keap1/Nrf2 signaling pathway, ultimately resulting in the expression of anti-oxidant enzymes. Keap1 protein negatively regulates Nrf2, and Nrf2 activation requires the oxidation of SH groups in Keap1 (45). HIIT decreases the Keap1 protein due to the increase in ROS, leading to the oxidation and modification of cysteine residues in Keap1. As a result, the degradation of the Nrf2 protein is prevented, and Nrf2 accumulates in the nucleus, inducing anti-oxidant gene expression (46-48). This finding is consistent with another study (49), which showed that 8 weeks of treadmill running at 60% maximum speed, 5 days a week, increased Nrf2 expression and anti-oxidant levels in rats. 

The levels of anti-oxidant enzymes, such as SOD, CAT, and GPx, also increased in the present study, which is in line with other studies (49, 50). Additionally, researchers (51) reported that regular physical activity can improve anti-oxidant defense in individuals with type 2 diabetes. Long-term high levels of ROS in individuals with diabetes can damage cellular macromolecules such as proteins, membrane lipids, and DNA. Extensive damage to these biomolecules in the brain can cause neuronal dysfunction and initiate apoptosis (52). Therefore, the increase in PGC1-α protects neurons from apoptosis by increasing the expression of anti-oxidant genes, and it also helps in maintaining synapses in the hippocampus. The present study also showed a reduction in the BAX/Bcl2 ratio after 8 weeks of HIIT and an increase in this ratio in T2D rats. An increase in the BAX/Bcl2 ratio indicates an increase in apoptosis, while a decrease in this ratio indicates a decrease in apoptosis. Hyperglycemia has been shown to cause high Bax protein expression by increasing free radicals (53). Previous studies (54-57) have reported a decrease in the BAX/Bcl2 ratio after exercise training. Bertram *et al*. (57) further reported that regular exercise can activate and strengthen the anti-oxidant defense system to compensate for adverse outcomes, such as neurodegeneration, apoptosis, and synapse damage resulting from increased oxidative stress and metabolic changes caused by type 2 diabetes. As the hippocampus plays a critical role in learning and memory (58), oxidative damage and increased apoptosis in this brain region can impair cognitive function (59). Therefore, maintaining the normal redox state in hippocampal neurons is crucial in preventing memory disorders.

## Conclusion

The present study indicates that a duration of eight weeks of high-intensity interval training (HIIT) can activate the Keap1-Nrf2 signaling pathway via an increase in PGC1α levels, resulting in increased anti-oxidant enzymes and resistance to oxidative stress, ultimately preventing apoptosis. Therefore, HIIT can potentially serve as a non-pharmacological approach to prevent and treat disorders associated with oxidative stress in diabetic brains. However, further clinical studies are necessary to validate these findings.

## Authors’ Contributions

K K conceived the study, contributed to methodology, writing, reviewing, and editing, supervised, performed project administration, and helped acquire funds; Sh N provided software assistance, formal analysis, data curation, and visualization; N E helped validate, investigate, and write the original draft; Z S acquired resources. 

## Supplementary Information

The datasets generated and/or analyzed during the current study are available in the Sara research lab repository, [https://www.sara-co.com/]. 

## Data Availability

The original contributions presented in the study are included in the article/supplementary material, and further inquiries can be directed to the corresponding author.

## Ethical Approval

This study was performed in line with the principles of the Declaration of Helsinki. Approval was granted by the Ethics Committee of Kerman University of Medical Sciences, Iran (IR.KMU.AEC.1401.003). 

## Conflicts of Interest

The authors have no relevant financial or non-financial interests to disclose.
